# Construction of a ZnO Heterogeneous Structure Using Co_3_O_4_ as a Co-Catalyst to Enhance Photoelectrochemical Performance

**DOI:** 10.3390/ma17010146

**Published:** 2023-12-27

**Authors:** Aiymkul A. Markhabayeva, Zhanar K. Kalkozova, Renata Nemkayeva, Yerassyl Yerlanuly, Assiya S. Anarova, Malika A. Tulegenova, Aida T. Tulegenova, Khabibulla A. Abdullin

**Affiliations:** Faculty of Physics and Technology, Al Farabi Kazakh National University, 71 Al-Farabi Avenue, Almaty 050040, Kazakhstan; zh.kalkozova@mail.ru (Z.K.K.); quasisensus@mail.ru (R.N.); yerlanuly@physics.kz (Y.Y.); assiya.anarova@gmail.com (A.S.A.); malika.tulegenova@bk.ru (M.A.T.); tulegenova.aida@gmail.com (A.T.T.); kh.abdullin@physics.kz (K.A.A.)

**Keywords:** photocatalyst, heterogeneous, nanorods, photoelectrochemical water splitting

## Abstract

Recently, heterostructured photocatalysts have gained significant attention in the field of photocatalysis due to their superior properties compared to single photocatalysts. One of the key advantages of heterostructured photocatalysts is their ability to enhance charge separation and broaden the absorption spectrum, thereby improving photocatalytic efficiency. Zinc oxide is a widely used n-type semiconductor with a proper photoelectrochemical activity. In this study, zinc oxide nanorod arrays were synthesized, and then the surfaces of ZnO nanorods were modified with the p-type semiconductor Co_3_O_4_ to create a p–n junction heterostructure. A significant increase in the photocurrent for the ZnO/Co_3_O_4_ composite, of 4.3 times, was found compared to pure ZnO. The dependence of the photocurrent on the morphology of the ZnO/Co_3_O_4_ composite allows for optimization of the morphology of the ZnO nanorod array to achieve improved photoelectrochemical performance. The results showed that the ZnO/Co_3_O_4_ heterostructure exhibited a photocurrent density of 3.46 mA/cm^2^, while bare ZnO demonstrated a photocurrent density of 0.8 mA/cm^2^ at 1.23 V. The results of this study provide a better understanding of the mechanism of charge separation and transfer in the heterostructural ZnO/Co_3_O_4_ photocatalytic system. Furthermore, the results will be useful for the design and optimization of photocatalytic systems for water splitting and other applications.

## 1. Introduction

In recent years, there has been a growing interest in alternative energy sources to compensate for diminishing fossil fuel resources. Harnessing solar energy is a promising way to counteract the energy crisis [1]. Efficient photoelectrochemical (PEC) water splitting systems offer a potential solution for the conversion of solar energy into other forms of energy. Effective water splitting technology requires a high light absorption coefficient and stable photocatalysts with appropriate bandgap energy. The energy levels of the conduction band (CB) in semiconductors need to be situated at a lower value than the hydrogen reduction potential (0 V vs. NHE), whereas the valence band (VB) should be at a higher energy level than the oxidation potential of oxygen (1.23 V vs. NHE). However, many metal oxide semiconductors that can absorb visible light and have a narrow bandgap of less than 2 eV do not have suitable VB and CB positions for water splitting. To overcome this, various techniques have been employed, such as doping with extraneous atoms to increase the number of states near the CB or VB, altering the particle size, using combinations of materials for efficient energy or electron transfer to absorb light with different energies, or employing plasmonic excitation to enhance the light absorption and photocatalytic efficiency [2,3,4,5].

Among the metal oxides, ZnO, TiO_2_ [6,7], and ZrO_2_ exhibit both conduction and valence band energies suitable for water splitting [8,9]. In particular, ZnO is an n-type direct-gap semiconductor with a band gap of ~3.3 eV at room temperature. The value of optical transmission is 85–90% in the range of 400–700 nm for ZnO thin films [10,11]. Due to its low toxicity and high electronic conductivity, it has been extensively studied for various optoelectronic applications [12], including solar cells, gas sensors, and phosphors. In addition, ZnO exhibits better light absorption than TiO_2_, because its refractive index is lower (2.0) than that of TiO_2_ (2.5–2.7), resulting in increased transparency and reduced light scattering.

However, zinc oxide is susceptible to photocorrosion in aqueous and acidic electrolytes and dissolves to form Zn(OH)_2_ [13]. Despite this limitation, experimental results have shown that ZnO actually exhibits higher photocatalytic activity than TiO_2_ and other semiconductor catalysts, such as CdS, WO_3_, Fe_2_O_3_, SnO_2_, ZrO_2_, and GaSe [14], especially in the degradation of dyes in aqueous solution [15,16,17,18,19]. These advantages make it a good candidate for a photoelectrode in photoelectrochemical cells for water oxidation.

Significant research efforts have been dedicated to the exploration of ZnO nanorod arrays, leading to the development of diverse device structures. This is evident in various reviews [20,21,22]. The appeal of such arrays lies in their facile synthesis, high crystallinity, excellent electrical properties, and substantial specific area. Noteworthy studies in related areas include an examination of morphological changes in a ZnO nanorod array with cobalt oxide particles, both pre- and post-photoelectrochemical (PEC) measurements [23]. Additionally, research, such as [24], has delved into variations in photoelectric properties in Co_3_O_4_/ZnO samples with different concentrations of the cobalt precursor. Another study, [25], focused on synthesizing ZnO/Co_3_O_4_ nanoparticles with varying cobalt contents. Considering these factors, the investigation of the photocatalytic properties of ZnO/Co_3_O_4_ structures for water decomposition emerges as a highly relevant and unexplored avenue in the existing body of literature.

The combination of ZnO with Co_3_O_4_, a p-type semiconductor with a band gap of 2.1 eV and known surface catalytic activity, showed improved photoelectrochemical performance [26] and oxygen evolution reaction [27], by separating charge carriers at the p–n interface [28,29]. Based on our previous experience [30], the formation of a WO_3_/Co_3_O_4_ p–n type heterojunction reduced the recombination of photocatalytic charges and enhanced the light absorption capabilities, resulting in a photocurrent 20 times higher than that of pristine WO_3_.

In this study, we synthesized a photocatalytic composite consisting of a ZnO nanorod array and a Co3O4 nanoparticle cocatalyst. A significant finding revealed a fourfold enhancement in the photocurrent within the ZnO/Co_3_O_4_ system when compared to ZnO alone. Furthermore, the photocurrent’s correlation with the size of ZnO nanorods exhibits a non-monotonic pattern, suggesting the potential for maximizing photocurrent and optimizing photocatalytic properties in the ZnO/Co_3_O_4_ system by tailoring the morphology of ZnO nanorod arrays.

## 2. Experimental Sections

### 2.1. Chemicals

Zinc acetate dehydrate (99.99%), zinc nitrate hexahydrate (98%), urotropine (99%), cobalt nitrate hexahydrate (99%), sodium sulfide (95%), and hexamethylenetetramine C_6_H_12_N_4_ (99%) were purchased from Sigma Aldrich (St. Louis, MO, USA). High purity water (18.2 MΩ·cm) was supplied via the ARIUM 611 DI water purification system, Sartorius Group (Göttingen, Germany). All reagents were used without purification.

### 2.2. Synthesis

#### 2.2.1. Spin Coating Technique for Deposition of ZnO Seed Layer

In the initial step, a spin coating technique was employed to deposit thin zinc oxide seed layers on the ITO substrates. Prior to synthesis, glass substrates coated with indium tin oxide (ITO) (resistivity of 10 Ω/cm) were thoroughly cleaned in a washing solution consisting of H_2_O:H_2_O_2_:NH_4_OH in a ratio of 4:1:1.

The sol was prepared by dissolving 0.4 g of zinc acetate Zn(CH_3_COO)_2_ in 10 mL of ethanol (96%) at room temperature. To ensure a clear solution, a few drops of lactic acid C_3_H_6_O_3_ (80%) were added to prevent aggregation. The resulting clear solution was then applied to the surface of the ITO substrate through centrifugation at 1000 rpm for 5 min.

Subsequently, the substrates were then dried at 100 °C for 15 min. A final annealing step was performed at 300 °C for 60 min in a muffle furnace. As a result, thin and transparent ZnO layers were formed on the ITO substrates.

#### 2.2.2. Chemical Bath Deposition of ZnO

ZnO nanorods were synthesized via chemical bath deposition. The second stage of the synthesis was the growth of ZnO nanorods. For this purpose, the substrates coated with seed layers were carefully placed into a beaker containing a solution composed of zinc nitrate, Zn(NO_3_)_2_, and hexamethylenetetramine, C_6_H_12_N_4_. The concentration of the solution was systematically varied at 25, 50, and 75 mM.

The beaker was then placed in a water bath and heated to a constant temperature of 90 °C. The reaction was allowed to proceed for 1 h. The substrates were then extracted from the solution, washed with distilled water, and dried at 100 °C. As a result of these procedures, ZnO nanorods were formed on the surface of the ITO substrate, demonstrating the successful outcome of this synthesis process.

#### 2.2.3. Deposition of Cobalt Oxide on the Surface of Zinc Oxide

A spin coating technique was further applied to deposit the cobalt oxide layer on top of the prepared ZnO nanorods. For sol preparation, cobalt nitrate, Co(NO_3_)_2_, was dissolved in 10 mL of ethanol at room temperature. ZnO photoelectrodes were fixed on a rotating disk, and then an alcoholic solution of cobalt nitrate was dropped onto the ZnO surface. The concentration of the cobalt solution was 0.007 M. The rotation speed was 1000 revolutions per minute (rpm) so that the solution completely covered the surface. The procedure was repeated three times, after which the samples were dried and then annealed at a temperature of 350 °C for 1 h in air. The rate of temperature increase during the heat treatment was 5 °C per minute.

## 3. Material Characterization

The film morphology was studied using scanning electron microscopy (SEM) on a Quanta 3D 200i microscope (FEI, Brno, Czech Republic) equipped with an energy dispersive X-ray spectroscopy (EDX) system. The crystal structure of the films was studied using a MiniFlex X-ray diffractometer (Rigaku, Tokyo, Japan). The XPS spectra were obtained using an X-ray photoelectron spectrometer (NEXSA, Thermo Scientific, Waltham, MA, USA). Raman spectra were obtained using a Ntegra Spectra (Solver Spectrum, NT-MDT Co., Ltd., Zelenograd, Russia) spectrometer with 473 nm excitation. Photocurrent measurements were performed using a xenon solar simulator (PLS-SXE300, Perfect light, China) with a 1.5 AM filter and a Corrtest CS310 potentiostat (Corrtest Instruments, China).

## 4. Photoelectrochemical Measurements

Photoelectrochemical (PEC) measurements including linear sweep voltammetry (LSV) and electrochemical impedance spectroscopy (EIS) were carried out to study the PEC material. Photocurrent measurements were performed using a Xenon solar lamp (AM 1.5 G, 100 mW/cm^2^), potentiostat, and a quartz cell. All photoelectrochemical and deposition experiments were carried out at room temperature using a standard three-electrode system with a Pt counter electrode and an Ag/AgCl reference electrode. The area of the working photoelectrode was 1 cm^2^, and the rest of the electrode was covered with epoxy resin. An aqueous solution of electrolyte containing Na_2_S (0.5 M) and Na_2_SO_3_ (0.5 M) was used as the electrolyte. The Nernst equation is used to convert potentials: $V_{RHE}=V_{Ag/AgCl}+0.0591\times pH+V_{Ag/AgCl}^{0}$, where V_Ag/AgCl_ is the applied potential, V^0^_Ag/AgCl_ is the standard potential of the Ag/AgCl reference electrode, and pH is basicity or acidity of the electrolyte. The pH of the electrolyte was 8.4. The sample was illuminated from the reverse side of the working electrode. The scan rate of the LSV under dark and light illumination was 10 mV/s.

## 5. Results and Discussion

**Figure 1:**
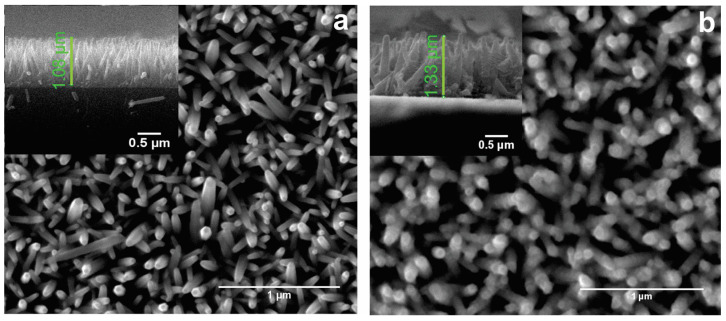
SEM images of ZnO nanorods grown from a solution with a zinc concentration of (**a**) 50 mM, (**b**) after cobalt deposition, and inset is cross-section images, respectively.

**Figure 2:**
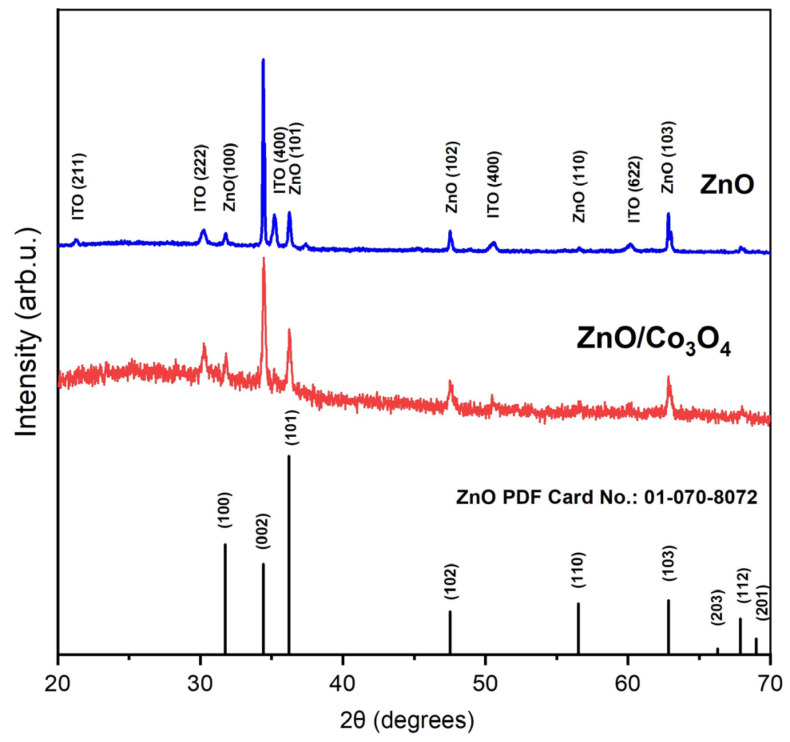
XRD patterns of ZnO and ZnO/Co_3_O_4_ on an ITO substrate.

Figure 1 illustrates the morphology of the samples obtained in this study. It can be seen (Figure 1) that the ZnO nanorods have a hexagonal shape and are predominantly located perpendicular to the substrate, and the ZnO (002) reflection in Figure 2 has the maximum intensity and corresponds to the preferential growth of nanorods; therefore, nanorods are single crystals. The surface of the ZnO nanorods (Figure 1a) is smooth; the surface becomes rough after cobalt deposition (Figure 1b). It is seen that the diameter and the length of nanorods is changed after cobalt deposition. Nevertheless, the Co_3_O_4_ layer exhibits uniform growth over ZnO nanostructures, forming an interconnected network of ultrathin Co_3_O_4_ that links all the ZnO nanostructures.

The diameter of the rods synthesized at different concentrations of zinc solution shows minimal variation (Figure S1). By examining the cross-sectional image, we can determine the length of these rods. The length of the nanorods and their diameter can be varied depending on the concentration of precursors in the growth solution at a fixed synthesis time. At a concentration of 25 mM, the rods have lateral length of approximately 530 nm (Figure S1a). At 50 mM, the lateral length increases to ~1 μm (Figure S1b). Finally, at 75 mM, the lateral length reaches ~1.5 μm (Figure S1c). These findings indicate that with increasing concentration, the diameters of the rods show minimal change, while the length of the rods increases significantly.

The following are the commonly referenced overall reactions in the literature for the synthesis of ZnO using zinc nitrate and hexamethylenetetramine (HMTA) precursors, as documented in references [31,32,33]:

(1) $${\left({CH}_{2}\right)}_{6}N_{4}+6H_{2}O\to 6HCHO+4NH_{3}$$

(2) $$NH_{3}+H_{2}O↔{NH}_{4}^{+}+OH^{-}$$

(3) $$2OH^{-}+{Zn}^{++}↔Zn{\left(OH\right)}_{2}$$

(4) $$Zn{\left(OH\right)}_{2}\to ZnO+H_{2}O$$

When HMTA is heated, it decomposes into ammonia and formaldehyde as shown in Equation (1). Subsequently, the ammonia reacts with water to generate hydroxide ions $OH^{-}$ (as depicted in Equation (2)), these hydroxide ions play a vital role in the crystallization of ZnO (Equations (3) and (4)). The concentration of precursors in a solution is a key factor in the growth process, as it directly influences the size of the resulting crystals. In the case of ZnO nanorods, the size-dependent properties of these structures have garnered significant attention due to their relevance in various applications [34,35,36]. For example, Lestari et al. showed that the structural and optical properties of ZnO nanorods varied with precursor concentration (0.002, 0.06, and 0.1 M) [37]. Horachit et al. fabricated a perovskite solar cell using ZnO nanorods, and the concentration of precursor solution (100 mM) affected the size and density of the nanorods, resulting in a 2.26% conversion efficiency [38]. Based on these findings, we selected precursor concentrations of 25, 50, and 75 mM to synthesize ZnO nanorods to study their impact on the photoelectrochemical properties.

X-ray diffraction studies of ZnO nanorods are shown in Figure 2. The peaks at angles of 21.2°, 30.2°, 35.15°, 50.6°, and 60.1° correspond to the ITO glass substrate. The diffraction peaks at 31.8°, 34.4°, 36.3°, 47.4°, 56.6°, and 62.8° can be indexed to the hexagonal phase of ZnO (PDF#01-0708072). A stronger peak intensity at the (002) plane compared to other peaks indicates the preferential growth of nanorods in the (002) direction. No significant changes were found in the XRD analysis after modification of ZnO with Co nitrate solution. This is explained by the low concentration of cobalt precursor in the heterojunction nanomaterial.

**Figure 3:**
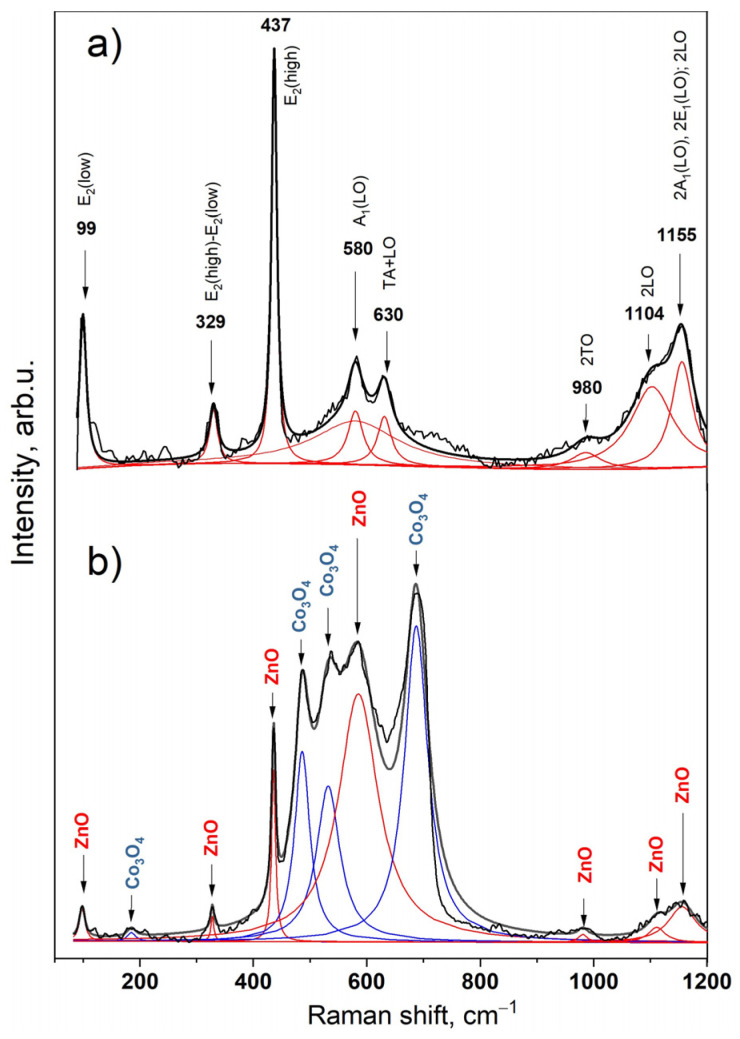
Raman spectra of (**a**) ZnO and (**b**) ZnO/Co_3_O_4_.

Raman spectroscopy was used to confirm the presence of cobalt oxide on top of zinc oxide. The Raman spectrum of the initial ZnO depicted in Figure 3a demonstrates the main typical bands at 99, 437, and 580 cm^−1^ assigned to E_2_ (low), E_2_ (high), and A_1_(LO)/E_1_(LO) phonon modes, respectively [39,40].

After the deposition of cobalt nitrate and annealing at 350 °C, the appearance of additional intense peaks corresponding to the Co_3_O_4_ phase are observed. The Raman spectrum of the ZnO/Co_3_O_4_ heterostructure with a combination of individual ZnO and Co_3_O_4_ bands is shown in Figure 3b. Cobalt oxide (Co_3_O_4_) in the spinel phase has a cubic structure with the main Raman active modes at 185, 486, 532, and 687 cm ^– 1^, which can be assigned to F_2g_, E_g_, F_2g_, and A_1g_ phonons due to the octahedral (CoO_6_) and tetrahedral (CO_4_) symmetry [41,42]. The assignments of all observed peaks of ZnO and ZnO/Co_3_O_4_ structures are presented in Table S1. The Raman spectra of the samples compared with the signal from the ITO glass substrate are shown in Figure S3 in the Supplementary Materials.

In addition, the presence of cobalt oxide was confirmed with energy-dispersive X-ray spectroscopy (EDS) analysis, the result of which is shown in Figure S2 for a pure ZnO sample. It is seen that there are no other elements except Zn and oxygen, and some low-intensity peaks corresponding to the substrate composition. The EDS result was able to confirm the presence of Co in the case of the ZnO/Co_3_O_4_ sample.

**Figure 4:**
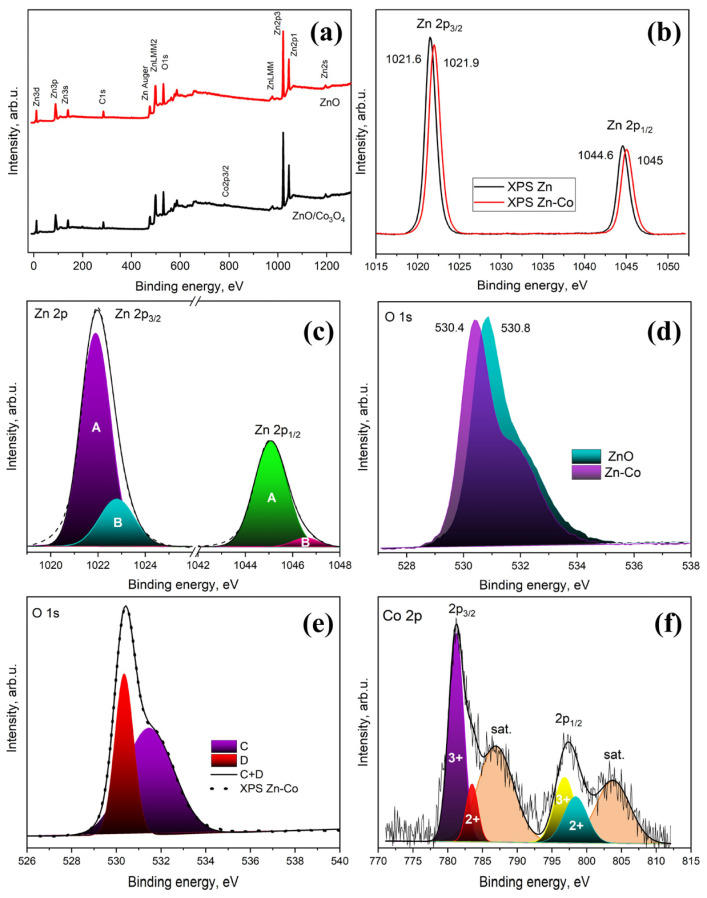
XPS survey of (**a**) ZnO and ZnO/Co_3_O_4_, (**b**) Zn 2p, (**c**) deconvolution of Zn 2p, (**d**) O 1s, (**e**) deconvolution of O 1s, and (**f**) Co 2p spectra.

In order to evaluate information from the surface region of the material, XPS analysis was also employed. Comparing the total XPS spectrum of ZnO and ZnO/Co_3_O_4_, we can observe an additional peak related with the cobalt (Co) core levels (Figure 4a).

Characteristic XPS peaks of Zn 2p at the binding energies of 1021.9 and 1045 eV confirm the atomic states Zn^2+^ 2p_3/2_ and Zn^2+^ 2p_1/2_, respectively. The Zn2p_3/2_ and Zn2p_1/2_ XPS peaks in the ZnO/Co_3_O_4_ samples are shifted upward in binding energy by 0.4 eV relative to the bare ZnO and have a larger half-width (Figure 4b).

These XPS Zn2p_3/2_ and Zn2p_1/2_ lines in the ZnO/Co_3_O_4_ samples can be represented as the sum of the XPS spectrum of the original ZnO and shifted to the right by 0.4 eV, with an intensity ratio of 1:4 (Figure 4c). The original spectrum belongs to ZnO atoms (Line A), in regions without Co_3_O_4_ particles. The spectrum shifted up in energy belongs to ZnO atoms, near which cobalt atoms are located (Line B).

Following the surface modification of ZnO with Co_3_O_4_, there was a noticeable shift of 0.5 eV toward higher binding energy in the O 1s peak as illustrated in Figure 4b. The O 1s peak can be divided into main peaks located at around 530 and 531 eV (Figure 4e). The presence of different metal cations can induce charge transfer effects leading to alterations in the electron density surrounding oxygen atoms and consequently influencing the position of the O 1s peak, as reported in references [43,44]. These findings suggest that the electronic cloud densities associated with surface Zn and O decrease as a result of some Co_3_O_4_ atoms bonding with them.

The Co 2p peak in the XPS spectrum typically exhibits multiple peaks corresponding to the different oxidation states of cobalt. The XPS bands Co2p_3/2_ and Co2p_1/2_ (Figure 4f) can be divided into two peaks: the peaks located at 781.9 eV and 797 eV correspond to Co^3+^, and the peaks at 785.5 eV and 798.4 eV correspond to Co^2+^. It can be seen that the Co^3+^ state has a higher concentration than Co^2+^, which indicates the predominance of the Co^3+^ composition (Figure 4f).

The ZnO/Co_3_O_4_ nanostructures’ photocatalytic efficiency was assessed by measuring the photocurrent response during exposure to simulated sunlight. Linear sweep voltammetry (LSV) was conducted both in the absence of light and under simulated solar illumination, with scans performed at a rate of 10 mV/s from 0 to 1V.

**Figure 5:**
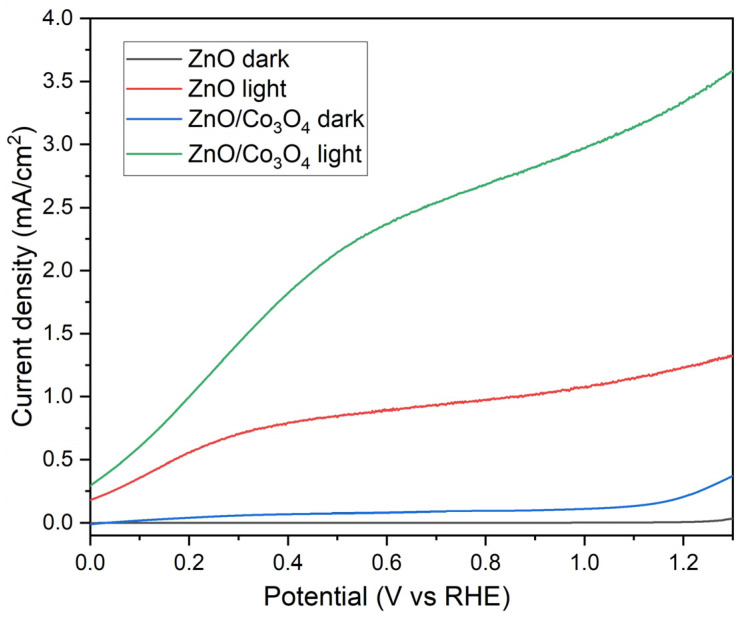
Comparison of LSV curves for bare ZnO nanorods and for ZnO/Co_3_O_4_ nanorods recorded at a scan rate of 10 mV/s in 0.5 M Na_2_SO_3_/Na_2_S electrolyte.

As expected, Figure 5a demonstrates that negligible current is observed under dark conditions. When we shine the light, all samples exhibit a substantial current response. We have tested ZnO nanorods deposited with 25, 50, and 75 mM concentrations to choose the best one to design a heterostructure and compared them. It is observed that the ZnO-50 mM sample shows higher values at all bias potential ranges (Figure S3). The ZnO-75 mM sample has the same value for current density of 1.6 mA/cm^2^ at 1 V potential; however, the current density at 0 V is lower compared to the ZnO-50 mM sample. This may be due to the appropriate diameter and density of ZnO nanorods deposited at the 50 mM concentration, which affects the light absorption behavior [45,46]. The dramatic increase in photocurrent in ZnO/Co_3_O_4_ compared to bare ZnO indicates that the contribution to the photocurrent comes from the p–n junction regions at the interface of ZnO and Co_3_O_4_. When such regions are larger, the photocurrent is greater. A ZnO array layer of nanorods with a 25 mM concentration has poor p–n junction regions. As the length of the nanorods increases at the 50 mM concentration, the specific area for p–n junctions increases, and the maximum value of the photocurrent is achieved. With a further increase in the thickness of the nanorod array layer at the 75 mM concentration, the conditions for collecting photogenerated carriers worsen due to an increase in the path of carriers to the current substrate. In addition, increasing the length of nanorods leads to shadowing, so increasing the length beyond the optimal one will not lead to an increase in photocurrent.

After optimizing the bare ZnO nanorods, we deposited cobalt nitrate solution and annealed it at 350 °C to design the ZnO/Co_3_O_4_ material. In Figure 5, a comparison of linear sweep voltammetry between bare ZnO and ZnO/Co_3_O_4_ is presented. The current value is significantly higher in the case of ZnO/Co_3_O_4_, owing to the synergistic effects that enhance charge generation, separation, and transport, as discussed in [26,27]. The introduction of Co_3_O_4_ has notably boosted the photocurrent throughout the entire potential range. Notably, at 1.23 V, there is a significant difference of 3.46 mA/cm^2^ between the dark and light current values, surpassing that of bare ZnO. This discrepancy underscores the synergistic effects and the improved electrochemical properties resulting from the incorporation of Co_3_O_4_.

**Figure 6:**
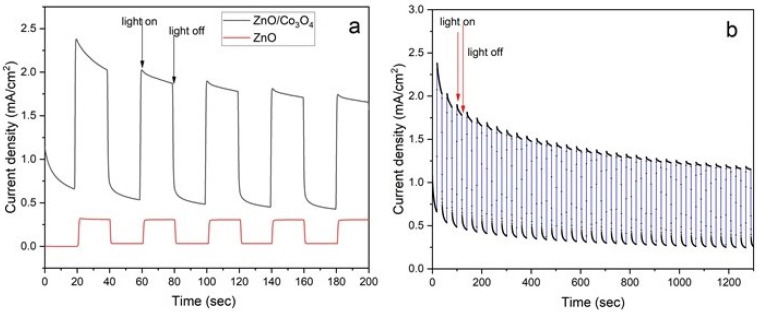
(**a**) Chronoamperometry I–t curves for the ZnO/Co_3_O_4_ compared with ZnO alone with an applied potential of 0.5 V versus RHE with 20 s light on/off cycles, and (**b**) ZnO/Co_3_O_4_ electrodes under choped illumination (100 mW/cm^2^) during 1300 s in 0.5 M Na_2_SO_3_/Na_2_S electrolyte.

We conducted a chronoamperometry experiment at 0.5 V, under chopped illumination, to compare the performance of ZnO/Co_3_O_4_ with that of pure ZnO. The photocurrent response was evaluated by alternately turning on and off simulated light, with each cycle lasting 20 s, as depicted in Figure 6a. ZnO/Co_3_O_4_ exhibits a much higher photocurrent than pristine ZnO. This aligns well with the findings from the LSV results. Chronoamperometries for the ZnO/Co_3_O_4_ electrodes obtained during 1300 s under chopped illumination are shown in Figure 6b. The j–t curve showing stability during 6000 s is shown in Figure S4. The current density initially drops to around 20%, but it subsequently displays an increasing trend compared to the initial value. This indicates the generation of new charge carriers and suggests that Co_3_O_4_ enhances the stability of the ZnO electrode. This observation further implies that the creation of a p–n junction within the ZnO/Co_3_O_4_ nanostructures effectively separates the photogenerated charge carriers, thereby reducing the recombination of photocatalytic charge carriers.

To investigate electron transfer dynamics at the interface between the photoelectrode and the electrolyte, we utilized electrochemical impedance spectroscopy (EIS) as illustrated in Figure S5. The reduced radius of the Nyquist plot is observed in the case of ZnO/Co_3_O_4_ photoelectrodes, when compared to ZnO, indicating improved charge transport capabilities.

**Figure 7:**
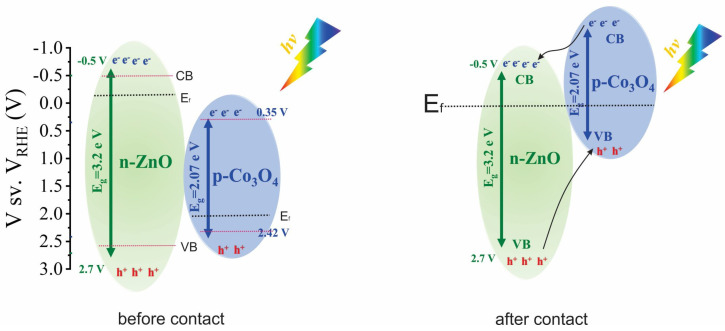
Schematic diagrams of energy bands of ZnO and Co_3_O_4_ and the charge separation process.

The conduction band energy positions of ZnO and Co_3_O_4_ are located at −0.5 eV and 0.35 eV, respectively. ZnO is an n-type semiconductor with a band gap of ~3.3 eV, and Co_3_O_4_ is a p-type semiconductor with a band gap of ~2.07 eV [47,48], as illustrated in Figure 7. When the ZnO/Co_3_O_4_ electrode is exposed to light, photogenerated electrons become excited, moving to the conduction band of ZnO, while holes are created in the VB of ZnO. Upon the contact of ZnO with Co_3_O_4_, there is a tendency for their Fermi levels to align, driven by the pursuit of equilibrium. This equilibration process involves the diffusion and drift of charge carriers between the two materials [49,50], resulting in the formation of a depletion layer at the interface. After equilibrium, the band diagram of Co_3_O_4_ is more shifted relative to ZnO; therefore, photogenerated electrons can move from Co_3_O_4_ to ZnO, while holes can move from the VB of ZnO to Co_3_O_4_ without any external bias potential (Figure 7).

**Figure 8:**
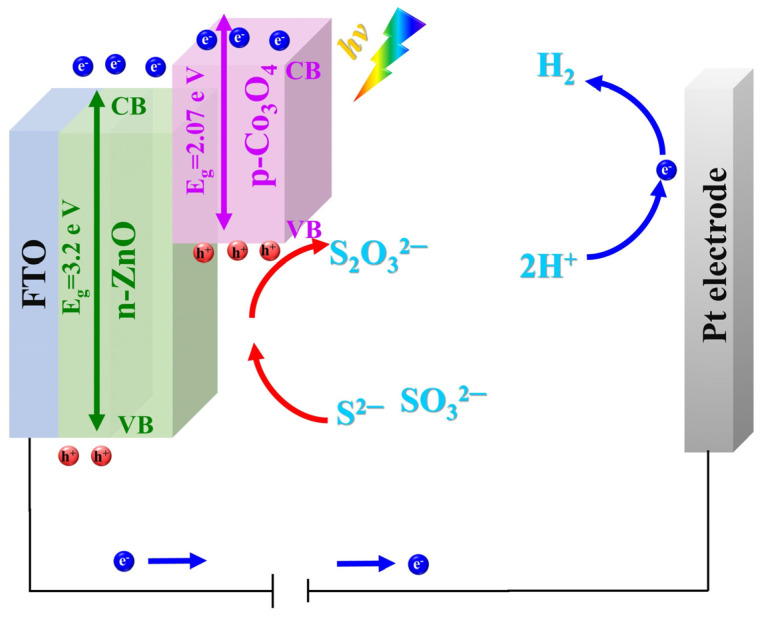
Schematic of the charge carrier’s transfer in a PEC cell.

Under a positive applied bias potential, as is typically the case, electrons have the ability to migrate towards the platinum (Pt) cathode, where they engage in the reduction reaction of water, as depicted in Figure 8. As a result, holes drift within the valence band (VB) and at the interface with the electrolyte. It is worth noting that in the case of bare ZnO, the process is primarily anodic photodecomposition rather than water oxidation.

The Na_2_S+Na_2_SO_3_ electrolyte is used as a sacrificial solution to reduce photocorrosion of the ZnO [51,52,53]. In this process, the $S^{2-}$ ions from Na_2_S can react with two holes in the VB of ZnO, forming $S_{2}^{2-}$. In addition, ${SO}_{3}^{2-}$ ions act as hole scavengers preventing a back reaction by reducing $S_{2}^{2-}$ to $S^{2-}$. These ${SO}_{3}^{2-}$ ions can also react with $S^{2-}$ to produce $S_{2}O_{3}^{2-}$. Consequently, the photogenerated holes oxidize Na_2_S + Na_2_SO_3_ electrolyte rather than water. This also improves the photocatalytic activity of ZnO-based heterostructures [54].

The ZnO/Co_3_O_4_ photocatalyst designed in our lab provides the highest photocurrent enhancement among some ZnO-based heterostructured photocatalysts with loading of other catalysts and other photocatalysts loaded with CoO_x_ as shown in Table 1.

**Figure 9:**
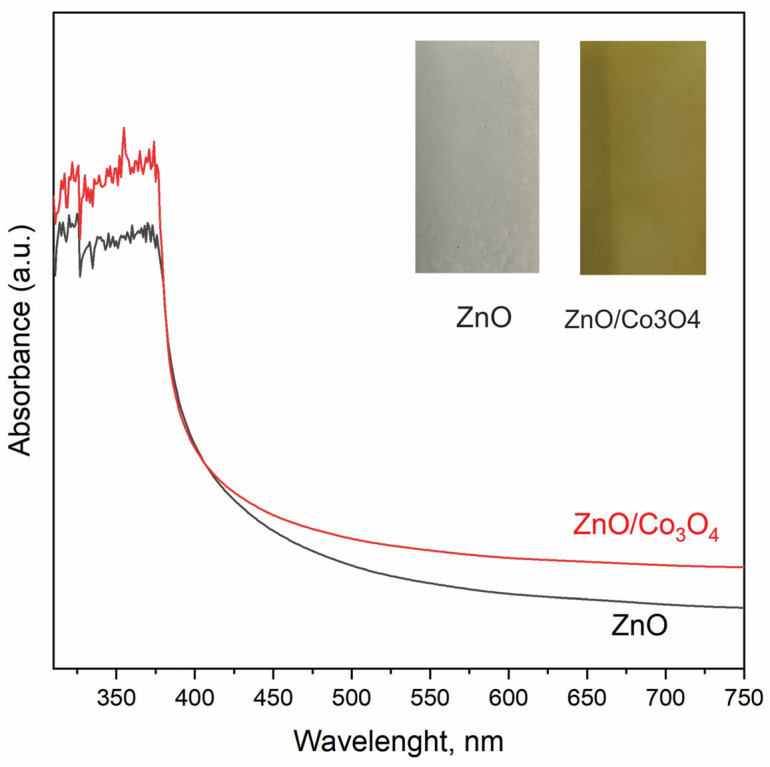
UV-vis absorption spectra of the bare ZnO and ZnO/Co_3_O_4_, the inset includes images of samples on the glass.

The optical characteristics of samples were examined through UV-vis absorption spectroscopy. In Figure 9, the UV-vis absorption spectra of both the bare ZnO and ZnO/Co_3_O_4_ heterostructures array are depicted. The photo of samples on the glass in the inset shows a transition from white to brown colors after cobalt deposition. The dominant absorption occurs in the UV region, around ~380 nm, characteristic of ZnO. It is apparent that the absorption in the visible region increases after cobalt deposition.

## 6. Conclusions

In conclusion, this research has demonstrated the successful utilization of a novel approach involving the combination of two different materials, namely ZnO nanorods as a photocatalyst and Co_3_O_4_ as a co-catalyst, achieved through chemical bath deposition (CBD) and a spin coating technique.

The solution concentration played a crucial role in the growth of zinc oxide nanorods, influencing their morphology, particularly the length of the nanorods. In this study, an optimal concentration of 50 mM was determined to be the best for efficient light absorption and enhancing photoelectrochemical properties.

Further improvement of PEC is realized by the formation of p–n junctions between ZnO and Co_3_O_4_ nanostructures. Notably, the photocurrent exhibited a significant value of 3.46 mA/cm^2^ at 1.23 V for ZnO/Co_3_O_4_ composite, while bare ZnO demonstrated a photocurrent density of 0.8 mA/cm^2^ at 1.23 V. These results underscore the potential of heterostructured photocatalysts as a promising area of research for the development of efficient photocatalysts and offer new insights into addressing the critical challenge of charge separation in water splitting.

## Figures and Tables

**Table 1:** Comparison of ZnO and other metal oxides combined with metal oxide nanomaterials.

Metal Oxides	Electrolyte	Light Source	Photocurrent Enhancement at 1.23 V vs. RHE (Ma/Cm^2^)	
ZnO/Co_3_O_4_	0.5 M Na_2_S/Na_2_SO_3_	Xe lamp 300 W	3.46	This work
ZnO/CoO	0.03 M borax	Xe lamp 300 W	1.26	[23]
ZnO/WO_3−x_	1 M Na_2_SO_4_	Xe arc lamp 300 W	2.39	[55]
ZnO NSs-NRs@TiO_2_-Au	0.5 M Na_2_SO_4_	-	1.73	[56]
ZnO@TiO_2_	0.1 M NaOH	Xe lamp 450 W	0.40	[57]
SnS_2_/b-ZnO	0.25 M Na_2_S/ 0.35 M Na_2_SO_3_	Xe arc lamp 300 W	0.58	[58]
Y-ZnO	0.1 M Na_2_SO_4_	Xe arc lamp 500 W	0.50	[59]

## Data Availability

The data presented in this study are available upon request from the corresponding author.

## Supplementary Materials

The following supporting information can be downloaded at: https://www.mdpi.com/article/10.3390/ma17010146/s1, Figure S1: SEM images of ZnO nanorods grown from a solution with a zinc concentration of (a) 25, (50), and 75 mM. The nanorod’s diameter obtained from SEM image grown from a solution with a zinc concentration of 25mM, 50mM, and 75mM zinc precursor solution; Figure S2: (a) EDS analysis for bare ZnO and (b) ZnO/Co_3_O_4_; Figure S3: Raman spectra of ZnO (left) and ZnO/ Co_3_O_4_ (right) samples along with Raman signal of ITO glass substrate; Figure S4: Comparison of LSV curves for bare ZnO nanorods with concentration of (a) 25, (b) 50 and 75 mM (c) recorded at scan rate of 10 mV/sec in 0.5 M Na_2_SO_3_/Na_2_S electrolyte; Figure S5: Chronoamperometry j-t curves for the ZnO/Co_3_O_4_ at an applied potential of 0.5 V versus RHE during 6000 s; Figure S6: Nyquist plots for bare ZnO and (b) ZnO/Co_3_O_4_; Table S1: Raman modes of ZnO and Co_3_O_4_.
