# Supplementary material for: Construction of a ZnO Heterogeneous Structure Using Co3O4 as a Co-Catalyst to Enhance Photoelectrochemical Performance
Source: Materials (Basel). 2023 Dec 27;17(1):146. doi: 10.3390/ma17010146 (PMC10779734; doi:10.3390/ma17010146)
Supplement: Supplementary file 1 [file materials-17-00146-s001.zip › materials-2724252-supplementary.pdf]

Supporting information for

Construction of a ZnO Heterogeneous Structure Using Co_3O_4 as a Co-Catalyst to Enhance Photoelectrochemical Performance

Aiymkul A. Markhabayeva *, Zhanar K. Kalkozova *, Renata Nemkayeva, Yerassyl Yerlanuly, Assiya A. Anarova, Malika A. Tulegenova, Aida T. Tulegenova and Khabibulla A. Abdullin

Al Farabi Kazakh National University, 71 Al-Farabi av., Almaty 050040, Kazakhstan; quasisensus@mail.ru (R.N.); yerlanuly@physics.kz (Y.Y.); assiya.anarova@gmail.com (A.A.A.); malika.tulegenova@bk.ru (M.A.T.); tulegenova.aida@gmail.com (A.T.T.); kh.abdullin@physics.kz (K.A.A.)

* Correspondence: aiko_marx@mail.ru (A.A.M.); zh.kalkozova@mail.ru (Z.K.K.)

1. Supplemental Figures and Tables

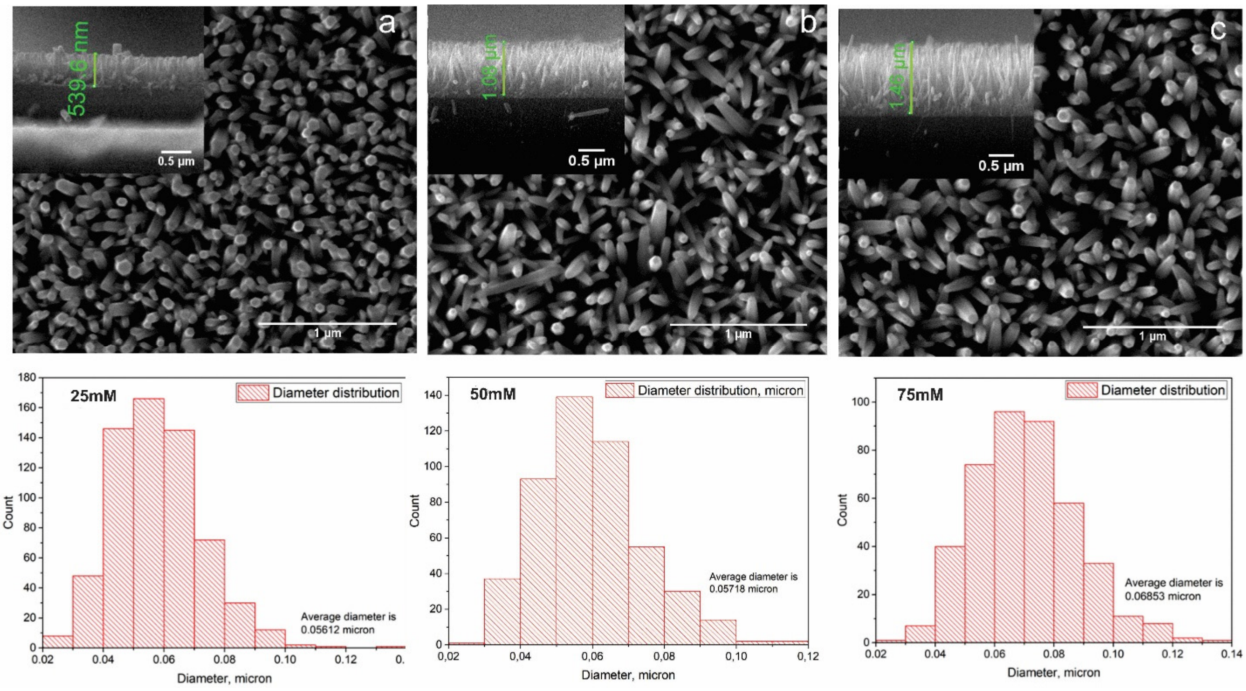

Figure S1. SEM images of ZnO nanorods grown from a solution with a zinc concentration of (a) 25, (50), and 75 mM. The nanorod's diameter obtained from SEM image grown from a solution with a zinc concentration of 25mM, 50mM, and 75mM zinc precursor solution.

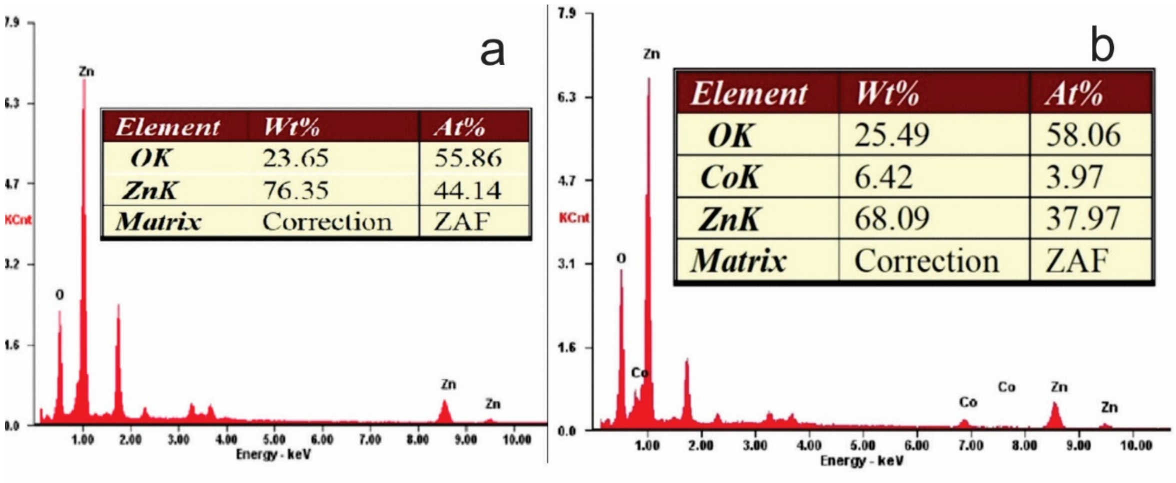

Figure S2. (a) EDS analysis for bare ZnO and (b) ZnO/Co_3O_4.

Table S1. – Raman modes of ZnO and Co_3O_4.

Peak position of ZnO (cm^{-1})	Assignment of mode	Peak position of Co_3O_4 (cm^{-1})	Assignment of mode
99	E_2(low)	185	F_{2g}
329	E_2(high)-E_2(low)	486	E_g
437	E_2(high)	532	F_{2g}
580	A_1(LO)/E_1(LO)	687	A_{1g}
630	TA+LO		
980	2TO		
1104	2LO		
1155	$2A_1$(LO), $2E_1$(LO)		

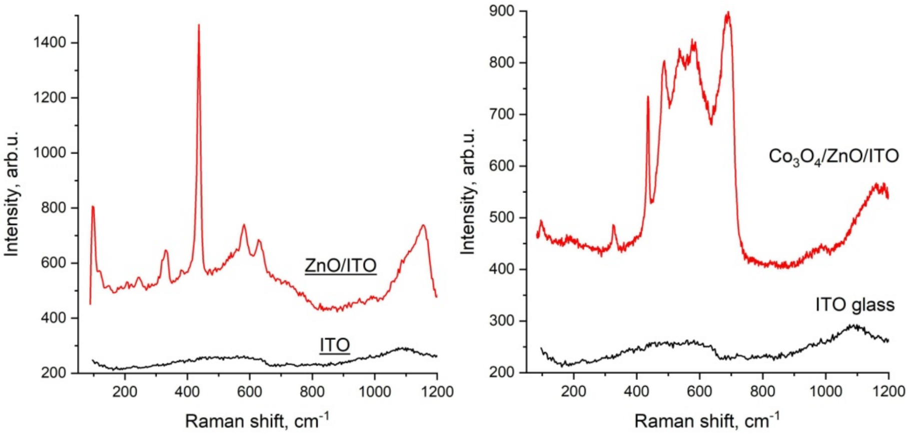

Figure S3. Raman spectra of ZnO (left) and ZnO/Co3O4 (right) samples along with Raman signal of ITO glass substrate.

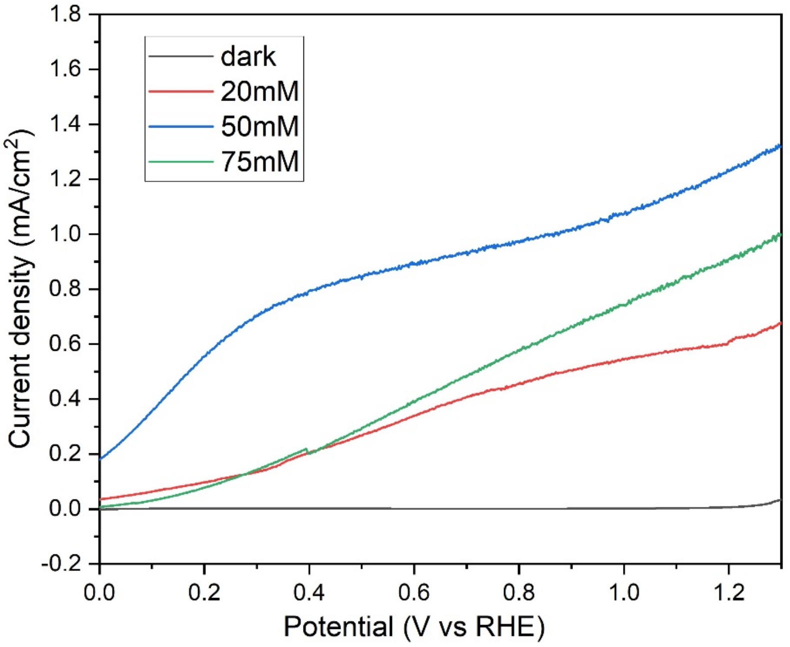

Figure S4. Comparison of LSV curves for bare ZnO nanorods with concentration of (a) 25, (b) 50 and 75 mM (c) recorded at scan rate of 10 mV/sec in 0.5 M Na_2SO_3/Na_2S electrolyte.

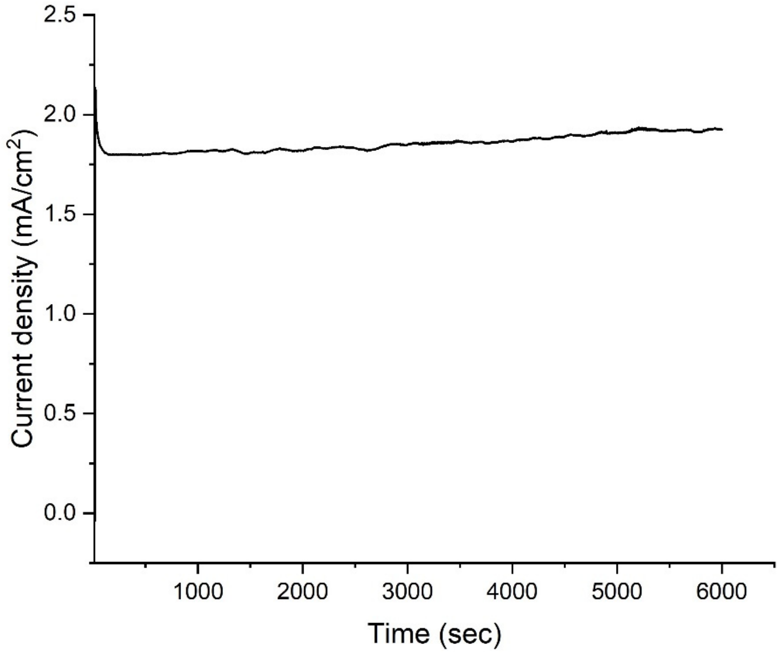

Figure S5. Chronoamperometry j-t curves for the ZnO/Co_3O_4 at an applied potential of 0.5 V versus RHE during 6000 sec.

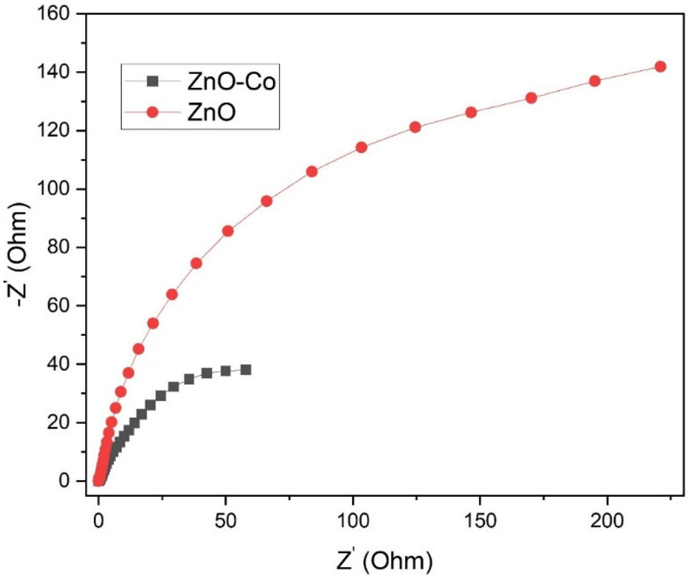

Figure S6. Nyquist plots for bare ZnO and (b) ZnO/Co_3O_4.
